# Supplementary material for: The beneficial effects of cumulus cells and oocyte-cumulus cell gap junctions depends on oocyte maturation and fertilization methods in mice
Source: PeerJ. 2016 Mar 3;4:e1761. doi: 10.7717/peerj.1761 (PMC4782716; doi:10.7717/peerj.1761)
Supplement: Supplemental Information 1 — Table S1. The role of cumulus cells and their gap junctions in oocyte maturation (n = 3). Table S2. Fertilization based on the number of GV oocytes (n = 3). Table S3. Fertilization based on the number of MII oocytes (n = 3). Table S4. Embryonic development based on the number of GV oocytes (n = 3). Table S5. Embryonic development based on the number of MII oocytes (n = 3). Table S6. Embryonic development based on number of 2-cell embryos (n = 3). [file peerj-04-1761-s002.docx]

**Supplemental tables**

**Table S1.** The role of cumulus cells and their gap junctions in oocyte maturation (n=3).

| Maturation | Abbreviation | PB1 % (mean ± SD) |
| --- | --- | --- |
| *In vivo* OCC | M-vivo-OCC | 98.78 ± 0.22^a^ |
| *In vitro* OCC | M-vitro-OCC | 97.57 ± 0.59^a^ |
| *In vitro* DO + cumulus | M-vitro-DC | 86.23 ± 4.05^b^ |
| *In vitro* DO | M-vitro-DO | 81.56 ± 6.48^b^ |

^a,b^Values with different superscripts within each column are significantly different (*P*< 0.05).

**Table S2.** Fertilization based on the number of GV oocytes (n=3).

| Maturation | Insemination | Abbreviation | 2-cell % (mean ± SD) |
| --- | --- | --- | --- |
| *In vivo* OCC | OCC | M-vivo-OCC + I-OCC | 91.68 ± 1.63^a^ |
|  | DO + cumulus | M-vivo-OCC + I-DC | 80.65 ± 2.32^b^ |
|  | DO | M-vivo-OCC + I-DO | 64.34 ± 2.30^c^ |
| *In vitro* OCC | OCC | M-vitro-OCC + I-OCC | 53.02 ± 8.00^d^ |
|  | DO + cumulus | M-vitro-OCC + I-DC | 55.72 ± 2.71^d^ |
|  | DO | M-vitro-OCC + I-DO | 45.62 ± 5.74^e^ |
| *In vitro* DO + cumulus | DO + cumulus | M-vitro-DC + I-DC | 46.62 ± 6.07^e^ |
|  | DO | M-vitro-DC + I-DO | 37.30 ± 2.50^f^ |
| *In vitro* DO | DO + cumulus | M-vitro-DO + I-DC | 47.15 ± 4.47^e^ |
|  | DO | M-vitro-DO + I-DO | 36.93 ± 5.29^f^ |

^a-f^Values with different superscripts within each column are significantly different (*P*< 0.05).

**Table S3.** Fertilization based on the number of MII oocytes (n=3).

| Maturation | Insemination | Abbreviation | 2-cell % (mean ± SD) |
| --- | --- | --- | --- |
| *In vivo* OCC | OCC | M-vivo-OCC + I-OCC | 92.86 ± 1.60^a^ |
|  | DO + cumulus | M-vivo-OCC + I-DC | 82.04 ± 2.06^b^ |
|  | DO | M-vivo-OCC + I-DO | 65.37 ± 2.60^c^ |
| *In vitro* OCC | OCC | M-vitro-OCC + I-OCC | 54.43 ± 7.94^d^ |
|  | DO + cumulus | M-vitro-OCC + I-DC | 56.75 ± 3.55^d^ |
|  | DO | M-vitro-OCC + I-DO | 46.39 ± 5.74^e^ |
| *In vitro* DO + cumulus | DO + cumulus | M-vitro-DC + I-DC | 53.98 ± 4.42^d^ |
|  | DO | M-vitro-DC + I-DO | 43.50 ± 4.48^e^ |
| *In vitro* DO | DO + cumulus | M-vitro-DO + I-DC | 57.95 ± 4.38^d^ |
|  | DO | M-vitro-DO + I-DO | 45.77 ± 6.48^e^ |

^a-e^Values with different superscripts within each column are significantly different (*P*< 0.05).

**Table S4.** Embryonic development based on the number of GV oocytes (n=3).

| Maturation | Insemination | Abbreviation | 4-cell %  (mean ± SD) | Morula %  (mean ± SD) | Blastocyst %  (mean ± SD) |
| --- | --- | --- | --- | --- | --- |
| *In vivo* OCC | OCC | M-vivo-OCC + I-OCC | 79.03 ± 4.73^a^ | 74.39 ± 2.48^a^ | 52.61 ± 6.08^a^ |
|  | DO + cumulus | M-vivo-OCC + I-DC | 62.38 ± 13.71^b^ | 52.26 ± 9.30^b^ | 36.01 ± 4.65^b^ |
|  | DO | M-vivo-OCC + I-DO | 27.47 ± 4.62^c^ | 22.55 ± 5.81^c^ | 15.30 ± 8.34^c^ |
| *In vitro* OCC | OCC | M-vitro-OCC + I-OCC | 21.72 ± 2.51^d^ | 17.26 ± 0.59^d^ | 7.66 ± 0.92^d^ |
|  | DO + cumulus | M-vitro-OCC + I-DC | 19.64 ± 4.89^d^ | 15.22 ± 1.89^de^ | 10.55 ± 1.18^d^ |
|  | DO | M-vitro-OCC + I-DO | 16.77 ± 2.03^d^ | 14.71 ± 1.32^de^ | 7.90 ± 2.02^d^ |
| *In vitro*  DO + cumulus | DO + cumulus | M-vitro-DC + I-DC | 18.01 ± 2.04^d^ | 14.41 ± 3.31^df^ | 6.22 ± 3.40^d^ |
|  | DO | M-vitro-DC + I-DO | 18.87 ± 1.58^d^ | 14.45 ± 1.50^df^ | 8.20 ± 0.92^d^ |
| *In vitro* DO | DO + cumulus | M-vitro-DO + I-DC | 17.16 ± 1.84^d^ | 12.03 ± 1.87^ef^ | 5.83 ± 0.69^d^ |
|  | DO | M-vitro-DO + I-DO | 11.90 ± 1.27^e^ | 10.41 ± 0.74^f^ | 5.97 ± 0.64^d^ |

^a-f^Values with different superscripts within each column are significantly different (*P*< 0.05).

**Table S5.** Embryonic development based on the number of MII oocytes (n=3).

| Maturation | Insemination | Abbreviation | 4-cell %  (mean ± SD) | Morula %  (mean ± SD) | Blastocyst %  (mean ± SD) |
| --- | --- | --- | --- | --- | --- |
| *In vivo* OCC | OCC | M-vivo-OCC + I-OCC | 80.06 ± 4.97^a^ | 75.35 ± 2.69^a^ | 53.29 ± 6.16^a^ |
|  | DO + cumulus | M-vivo-OCC + I-DC | 63.42 ± 13.69^b^ | 53.14 ± 9.25^b^ | 36.65 ± 4.85^b^ |
|  | DO | M-vivo-OCC + I-DO | 27.92 ± 4.84^c^ | 22.93 ± 6.03^c^ | 15.57 ± 8.56^c^ |
| *In vitro* OCC | OCC | M-vitro-OCC + I-OCC | 22.32 ± 2.68^d^ | 17.73 ± 0.59^d^ | 7.88 ± 0.99^d^ |
|  | DO + cumulus | M-vitro-OCC + I-DC | 19.98 ± 3.35^d^ | 15.49 ± 1.19^d^ | 10.77 ± 2.77^d^ |
|  | DO | M-vitro-OCC + I-DO | 17.04 ± 1.84^de^ | 14.95 ± 1.20^d^ | 8.05 ± 2.12^d^ |
| *In vitro*  DO + cumulus | DO + cumulus | M-vitro-DC + I-DC | 20.93 ± 2.46^d^ | 16.80 ± 4.30^d^ | 7.33 ± 4.18^d^ |
|  | DO | M-vitro-DC + I-DO | 21.96 ± 1.98^d^ | 16.78 ± 1.14^d^ | 9.57 ± 1.41^d^ |
| *In vitro* DO | DO + cumulus | M-vitro-DO + I-DC | 21.03 ± 0.65^d^ | 14.83 ± 2.46^d^ | 7.21 ± 1.28^d^ |
|  | DO | M-vitro-DO + I-DO | 14.72 ± 0.90^e^ | 12.95 ± 1.53^d^ | 7.41 ± 0.85^d^ |

^a-e^Values with different superscripts within each column are significantly different (*P*< 0.05).

**Table S6.** Embryonic development based on number of 2-cell embryos (n=3).

| Maturation | Insemination | Abbreviation | 4-cell %  (mean ± SD) | Morula %  (mean ± SD) | Blastocyst %  (mean ± SD) |
| --- | --- | --- | --- | --- | --- |
| *In vivo* OCC | OCC | M-vivo-OCC + I-OCC | 86.23 ± 5.71^a^ | 86.23 ± 5.71^a^ | 57.32 ± 5.64^a^ |
|  | DO + cumulus | M-vivo-OCC + I-DC | 77.06 ± 14.54^b^ | 64.62 ± 9.56^b^ | 44.78 ± 6.93^b^ |
|  | DO | M-vivo-OCC + I-DO | 42.61 ± 6.23^cd^ | 34.95 ± 8.33^c^ | 23.56 ± 12.44^c^ |
| *In vitro* OCC | OCC | M-vitro-OCC + I-OCC | 42.10 ± 11.57^de^ | 33.09 ± 5.40^cde^ | 14.86 ± 4.20^d^ |
|  | DO + cumulus | M-vitro-OCC + I-DC | 35.42 ± 7.41^de^ | 27.39 ± 3.32^de^ | 18.95 ± 4.67^d^ |
|  | DO | M-vitro-OCC + I-DO | 36.87 ± 3.13^de^ | 32.38 ± 2.03^cde^ | 17.20 ± 2.82^de^ |
| *In vitro*  DO + cumulus | DO + cumulus | M-vitro-DC + I-DC | 39.05 ± 6.75^de^ | 31.59 ± 10.15^cde^ | 13.99 ± 8.74^d^ |
|  | DO | M-vitro-DC + I-DO | 50.80 ± 6.17^c^ | 39.03 ± 6.79^cd^ | 21.94 ± 1.28^ce^ |
| *In vitro* DO | DO + cumulus | M-vitro-DO + I-DC | 36.47 ± 3.70^e^ | 25.84 ± 5.71^e^ | 12.52 ± 2.55^d^ |
|  | DO | M-vitro-DO + I-DO | 32.74 ± 6.15^e^ | 28.76 ± 6.11^e^ | 16.58 ± 4.32^de^ |

^a-e^Values with different superscripts within each column are significantly different (*P*< 0.05).
